# Hypoglycemic and antilipidemic properties of kombucha tea in alloxan-induced diabetic rats

**DOI:** 10.1186/1472-6882-12-63

**Published:** 2012-05-16

**Authors:** Ahmed Aloulou, Khaled Hamden, Dhouha Elloumi, Madiha Bou Ali, Khaoula Hargafi, Bassem Jaouadi, Fatma Ayadi, Abdelfattah Elfeki, Emna Ammar

**Affiliations:** 1 Laboratory of Biochemistry and Enzymatic Engineering of Lipases, National School of Engineers of Sfax, University of Sfax, Sfax, 3038, Tunisia; 2 Biotechnology High School of Sfax, University of Sfax, Sfax, 3052, Tunisia; 3 Research Unit Management of Coastal and Urban environments, National School of Engineers of Sfax, University of Sfax, Sfax, 3038, Tunisia; 4 Research Unit Molecular Bases of Human Diseases, Sfax College of Medicine, University of Sfax, Sfax, 3000, Tunisia; 5 Laboratory of Microorganisms and Biomolecules, Centre of Biotechnology of Sfax, University of Sfax, Sfax, 3018, Tunisia

## Abstract

**Background:**

Diabetes has become a serious health problem and a major risk factor associated with troublesome health complications, such as metabolism disorders and liver-kidney dysfunctions. The inadequacies associated with conventional medicines have led to a determined search for alternative natural therapeutic agents. The present study aimed to investigate and compare the hypoglycemic and antilipidemic effects of kombucha and black tea, two natural drinks commonly consumed around the world, in surviving diabetic rats.

**Methods:**

Alloxan diabetic rats were orally supplied with kombucha and black tea at a dose of 5 mL/kg body weight per day for 30 days, fasted overnight, and sacrificed on the 31st day of the experiment. Their bloods were collected and submitted to various biochemical measurements, including blood glucose, cholesterol, triglcerides, urea, creatinine, transaminases, transpeptidase, lipase, and amylase activities. Their pancreases were isolated and processed to measure lipase and α-amylase activities and to perform histological analysis.

**Results:**

The findings revealed that, compared to black tea, kombucha tea was a better inhibitor of α-amylase and lipase activities in the plasma and pancreas and a better suppressor of increased blood glucose levels. Interestingly, kombucha was noted to induce a marked delay in the absorption of LDL-cholesterol and triglycerides and a significant increase in HDL-cholesterol. Histological analyses also showed that it exerted an ameliorative action on the pancreases and efficiently protected the liver-kidney functions of diabetic rats, evidenced by significant decreases in aspartate transaminase, alanine transaminase, and gamma-glytamyl transpeptidase activities in the plasma, as well as in the creatinine and urea contents.

**Conclusions:**

The findings revealed that kombucha tea administration induced attractive curative effects on diabetic rats, particularly in terms of liver-kidney functions. Kombucha tea can, therefore, be considered as a potential strong candidate for future application as a functional supplement for the treatment and prevention of diabetes.

## Background

Diabetes mellitus (DM) is a chronic metabolic disorder that constitutes a major public health problem throughout the world. Current estimates indicate that approximately 4% of the global population suffer from DM, a percentage which is expected to reach 5.4% in 2025 [1]. This disease is a multifactor disorder associated with chronic hyperglycemia and troublesome disruptions in carbohydrate, fat, and protein metabolisms emanating from deficiencies or disruptions in insulin secretion [2], defects in reactive oxygen species scavenging enzymes [3], and high oxidative stress impairing pancreatic beta cells [4,5]. Hyperglycemia leads to long-term tissue damages and complications, such as liver-kidney dysfunctions, often associated with serious diseases [6,7].

The prevalence of type 2 diabetes mellitus is increasing worldwide at alarming rates. Several therapeutic strategies are currently available for the treatment of this chronic metabolic disorder, including the stimulation of endogenous insulin secretion, enhancement of insulin action at the target tissues, inhibition of dietary starch and lipid degradation, and treatment with oral hypoglycemic agents [8]. The limitations associated with those therapeutic strategies have led to a determined search for more efficient and cost-effective alternatives. This trend has been further intensified by increasing doubts surrounding current dietary and other lifestyle behaviors together with growing interests in functional foods and nutraceuticals [9]. Complementary and alternative medicine applications have attracted special attention in recent research for they offer new promising opportunities for the development of efficient, side effect-free, and lower cost alternatives to existing synthetic hypoglycemic agents [10-12].

Of particular relevance to this argument, kombucha tea (KT), a traditional drink made from a particular fermentation of sugared black tea (BT) and a symbiosis of yeast species, fungi, and acetic acid bacteria, is commonly consumed throughout the world as a medicinal health-promoting beverage [13]. Although the beneficial and/or adverse effects of kombucha tea on human health have not been scientifically determined yet, there are several reasons to believe that kombucha may have desirable positive effects on human health. In fact, the metabolic and health effects of several probiotic products are gathering increasing momentum in recent years. A number of currently commercialized food products (e.g. yogurt, cheese, fermented vegetables and kefir) are known to contain live bacteria, or metabolites of bacteria, produced during similar fermentation processes, and are considered as health promoting probiotic foodstuffs [14,15]. Moreover, several studies have recently demonstrated that kombucha can reduce cell damage induced by oxidative stress [16-20]. Kombucha has also been reported to constitute a potent therapeutic supplement that improved resistance against cancer, prevented cardiovascular diseases, promoted digestive functions, stimulated the immune system, and reduced inflammatory problems [17,21-23].

Tea and kombucha are presented in the literature as two very distinct beverages and no correlation has so far been reported between them [24]. Some of the effects reported for kombucha intakes are, however, very similar to those described for tea [24]. Nevertheless, while the composition, properties, and effects of tea on chronic and progressive illnesses, such as diabetes, are well documented in the literature [25], little data are currently available on these issues with regards to kombucha. In fact, most of the data on kombucha tea is anecdotal and further studies are needed to elucidate its putative therapeutic potential, particularly against DM.

In this context, pancreatic lipase, a complex enzyme that plays a key role in lipid metabolism, has often been employed in human and animal model studies involving the evaluation of natural products for potential application as antiobesity and antidiabetic agents [8]. The inhibition of this enzyme significantly decreases the digestion and uptake of lipids, thereby decreasing the level of postprandial blood glucose in non-insulin-dependent diabetic patients. Pancreatic α-amylase is a key enzyme in the digestive system that catalyses the initial step in the hydrolysis of starch to a mixture of smaller oligosaccharides consisting of maltose, maltotriose, and a number of oligoglucans. These are then acted on by α-glucosidases and further degraded to glucose that enters the blood-stream. The degradation of this dietary starch proceeds rapidly and leads to elevated postprandial hyperglycemia. Inhibitors of pancreatic α-amylase delay carbohydrate digestion, thus reducing glucose absorption rates and lowering postprandial serum glucose levels [26].

Considering the increasing concerns over the alarming rates recorded for DM and in light of the promising opportunities that kombucha might open with regards to the alleviation and/or prevention of this troublesome disease, the present study was undertaken to investigate and assess the hypoglycemic and antilipidemic effects of kombucha using a diabetic rat model. It aims to gain principled insights with regards to the biological activities of kombucha towards pancreatic lipase and α-amylase as well as its effects on liver-kidney function, which may provide a starting point for the understanding of the antidiabetic potential of kombucha. For the sake of pertinence, the biological activities of kombucha were compared to those reported for black tea [27].

## Methods

### Black tea preparation

One hundred grams of sugar were added to 1 L of distilled water, and the solution was boiled for 15 min in a sterile conical flask. Black tea powder (Lipton) was added to the flask (12 g/L), which was then left to cool down at room temperature for one hour. The mixture was filtered using a sterile nylon mesh, and the filtrate was used as black tea [17].

### Kombucha preparation

The kombucha cultures employed in the present work were purchased from a French commercial online outlet (Kombucha-Shop). They are known as symbionts of yeasts (e.g. *ZygosaccharomycesSchizosaccharomycesTorulosporaRhodotorulaBrettanomycesCandidaPichia*, and *Zygosaccharomyces*) and bacteria (e.g. *Acetobacter*) [17,28]. The cultures were stored at 4°C prior to fermentation. Black tea was poured into 1-L glass jars, which were sterilized beforehand, and inoculated with 2.5% (w/v) of freshly grown kombucha mat that had been grown and maintained in the same medium [29]. The fermentation, kept under aseptic conditions, was carried out by incubating the kombucha culture at 28 ± 1°C for 12 days. The flask was covered with clean cheese cloth and fixed with rubber bands. The medium (brew) was then centrifuged aseptically at 1500 × g for 30 min and stored in polypropylene vials at −20°C for further use [17]. New kombucha mat developed over the mother culture.

### Animals and treatments

The experimental protocols and procedures used in the present work were approved by the Ethics Committee of the University of Sfax (Sfax, Tunisia) for the care and use of laboratory animals. Adult male Wistar rats, weighing 179 ± 10 g, were obtained from the Central Pharmacy (Tunisia). The animals were kept in an environmentally controlled breeding room where they had free access to tap water and pellet diet (Sico, Tunisia) [30].

### Induction of diabetes

Diabetes was induced through a single intraperitoneal injection of a freshly prepared alloxan (Sigma-Aldrich, USA) solution in normal saline at a dose of 150 mg/kg body weight. Since the injection of alloxan can provoke fatal hypoglycemia due to a reactive massive release of pancreatic insulin, the rats were also orally given 5–10 ml of a 20% glucose solution after 6 h. The animals were then kept with free access to 5% glucose solution for the next 24 h to prevent severe hypoglycemia [31]. Two weeks later, the rats with moderate diabetes having glycosuria and hyperglycemia (i.e. with blood glucose levels of ≥2 g/l) were chosen for the experiments. They were fasted overnight before being sacrificed by decapitation for blood and tissue analyses.

### Experimental design

The rats were subdivided into six experimental groups of eight animals each. Each group was submitted to a specific treatment as follows. Normal control and diabetic rats, referred to as [Con] and [Diab] groups, respectively, were fed with normal diet and drinking water *ad libitum*. Diabetic rats that received KT and BT by gastric gavage (5 ml per kg of body weight) every day [5] were designated as [Diab + KT] and [Diab + BT] groups, respectively. Normal rats that were given KT and BT by gastric gavage (5 ml per kg of body weight) every day were termed as [Con + KT] and [Con + BT] groups, respectively.

One month later, the rats were weighed and sacrificed by decapitation, and their trunk bloods were collected. Plasma was immediately separated by centrifugation at 4°C and 1500 × g for 15 min. The pancreases were removed and trimmed free of fat. The samples were stored at −80°C until further use.

### Biochemical study

The pancreas of each rat was excised and then homogenized and centrifuged (5000 × g, 20 min, 4°C). The supernatant was frozen and stored till further use in subsequent enzymatic assays. Protein content was estimated by the method described by Lowry *et al.*[32]. The activities of aspartate transaminase [26], alanine transaminase (ALT), gamma-glutamyl transpeptidase (GGT), and α-amylase as well as the levels of glucose, urea, creatinine, total cholesterol, triglycerides (TG), and high-density lipoprotein-cholesterol (HDL-Ch) in the serum were measured using commercial kits from Biomaghreb Analyticals (Tunisia). All assessment assays and kits were performed in accordance with the manufacturers’ instructions and protocols. Lipase activity was measured on tributyrin using a pH-stat titrator at pH 8 and 37°C as previously described elsewhere [33].

### Histological study

For histological analyses, pieces of pancreas were fixed in a Bouin solution for 24 h and then embedded in paraffin. Sections of 5 μm thickness were stained with hematoxylin-eosin and examined under an Olympus CX41 light microscope [34,35].

### Statistical analysis

Statistical analysis was performed using the Statistical Package for the Social Sciences (SPSS, Version 10.0). Data are presented as means ± SD. Determinations were obtained from eight animals per group, and the differences were examined using one-way analysis of variance (ANOVA) followed by the Fisher test (Stat View). Statistical significance was accepted at p < 0.05.

## Results

### Effect of kombucha on the pancreatic tissue architecture of diabetic rats

**Figure 1:**
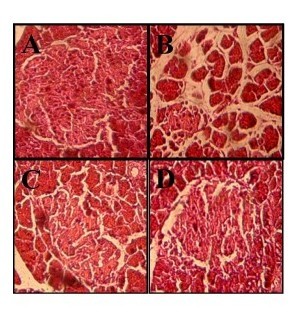
**Effect of KT and BT on the histological changes of the pancreas of the rats evaluated by haematoxylin and eosin (H&E) staining (100×).** (**A**) Normal control rats (Con) showed normal β cells. (**B**) Severe injury in the β-Cells of the pancreas of diabetic control rats (Diab). (**C**) and (**D**) ameliorative action of KT and BT on the architecture of the pancreas of diabetic rats treated with BT and KT, respectively.

The findings from the histopathological analyses revealed that while the pancreatic tissues of the control rats (Figure 1A) exhibited normal islets, those of the alloxan-induced diabetic rats showed clear atrophy of β-Cells (Figure 1B). The pancreas of the diabetic rats that were treated either with kombucha or black teas were, on the other hand, noted to undergo a marked amelioration (Figure 1C and 1D). Furthermore, the architectures of the pancreas of the normal rats treated with the kombucha and black teas were noted to be similar to that of the normal control rats (data not shown).

### Plasma and pancreas α-amylase activity and plasma glucose level

**Figure 2:**
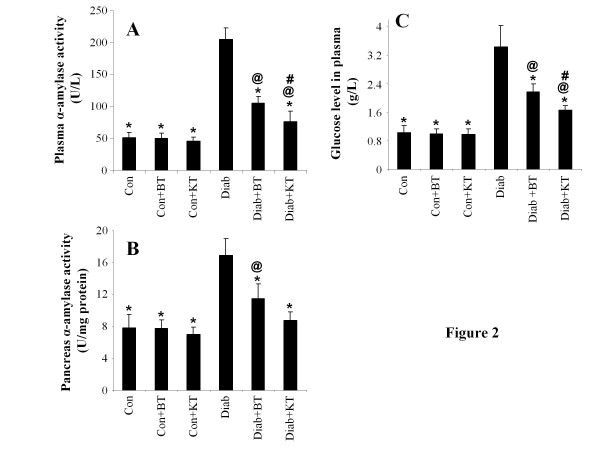
**Effect of KT and BT on α-amylase activity in plasma (A), pancreas (B), and blood glucose levels (C) of surviving diabetic rats.** Data represent mean ± S.D (n = 8 for each group). The values are statistically significant and presented as follows: single asterisk (*), p < 0.05 vs. control diabetic rats (Diab); commercial at (@), p < 0.05 vs. respective control rats (Con) [i.e. on the same corresponding tea treatment]; number sign (#), p < 0.05 vs. diabetic rats treated with BT (Diab + BT).

Figure 2 shows that, compared to the their counterparts from the normal control group, the rats from the control diabetic rats underwent significant increases in terms of plasma and pancreas α-amylase activities, which reached up to 405 ± 53% (p < 0.05) and 225 ± 52% (p < 0.05), respectively. The black tea supplement administered to diabetic rats was, however, noted to bring about a significant decrease in the plasma and pancreas α-amylase activities, reaching to 52 ± 11% (p < 0.05) and 70 ± 17% (p < 0.05), when compared to untreated diabetic rats. This inhibitory effect of black tea on α-amylase activity was followed by a decrease in the rate of blood glucose that reached up to 65 ± 14% (p < 0.05) (Figure 2C). Considerable α-amylase activity reductions of up to 37 ± 8% (p < 0.05) and 52 ± 7% (p < 0.05) were also observed in the plasma and pancreas of the diabetic rats treated with kombucha, respectively, as compared to those of the untreated diabetic rats. The kombucha supplement was also observed to bring about a significant decrease of 50 ± 11% in terms of blood glucose concentration (p < 0.05) (Figure 2C).

### Plasma and pancreas lipase activity and plasma lipids concentration

**Figure 3:**
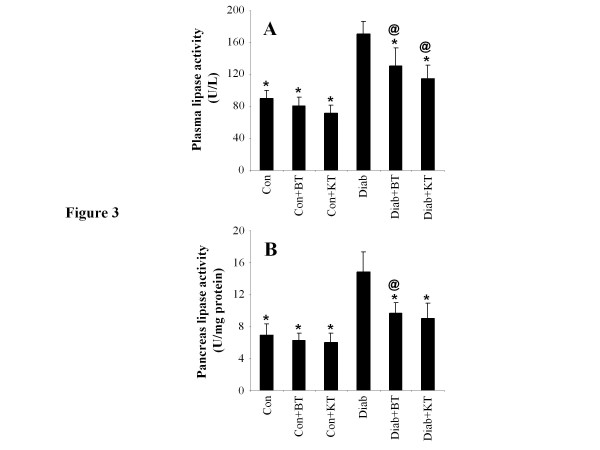
**Effect of KT and BT on lipase activity in plasma (A) and pancreas (B) of surviving diabetic rats.** Data represent mean ± S.D (n = 8 for each group). The values are statistically significant and presented as follows: single asterisk (*), p < 0.05 vs. control diabetic rats (Diab); commercial at (@), p < 0.05 vs. respective control rats (Con) [i.e. on the same corresponding tea treatment]; number sign (#), p < 0.05 vs. diabetic rats treated with BT (Diab + BT).

**Figure 4:**
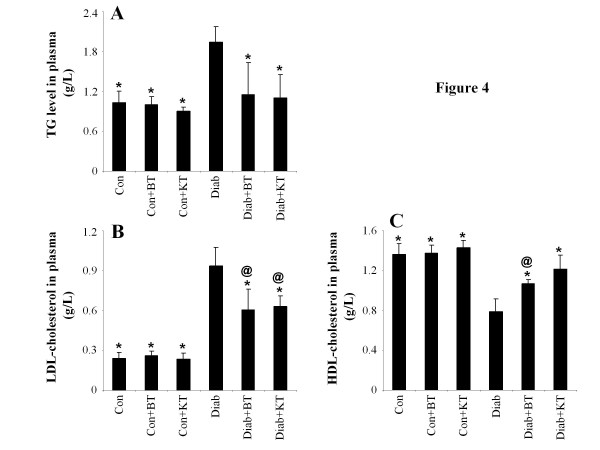
**Effect of KT and BT on TG (A), LDL-Ch (B), and HDL-Ch (C) levels in plasma of surviving diabetic rats.** Data represent mean ± S.D (n = 8 for each group). The values are statistically significant and presented as follows: single asterisk (*), p < 0.05 vs. control diabetic rats (Diab); commercial at (@), p < 0.05 vs. respective control rats (Con) [i.e. on the same corresponding tea treatment]; number sign (#), p < 0.05 vs. diabetic rats treated with BT (Diab + BT).

Figure 3 indicates that, when compared to that of non-diabetic rats, the lipase activity in both the plasma and pancreas of diabetic rats underwent significant (p < 0.05) increases of up to 194 ± 35% and 220 ± 41% respectively. This increase in lipase activity stimulated lipid absorption and, consequently, led to significant (p < 0.05) increases of 194 ± 50% and 404 ± 107% (p < 0.05) in the TG and low-density lipoprotein-cholesterol (LDL-Ch) concentrations in the plasma, respectively, as compared to non-diabetic rats (Figure 4). The administration of black tea to diabetic rats was, on the other hand, noted to induce significant (p < 0.05) reductions of 80 ± 15% and 68 ± 17% (p < 0.05) in terms of lipase activity in the plasma and pancreas and of 59 ± 21% and 65 ± 14% in terms of blood TG and LDL-Ch levels, respectively, again as compared to those of the untreated diabetic rats. The administration of kombucha to surviving diabetic rats was also observed to have reverted the activity of lipase in plasma and pancreas back to 68 ± 10% and 62 ± 10% (p < 0.05), respectively. This supplement was, also, observed to bring about a considerable decrease in LDL-Ch and TG concentrations in the plasma. Moreover, while diabetes was noted to induce a significant (p < 0.05) decrease of 58 ± 12% in the plasma HDL-Ch concentration, black tea and kombucha supplements were observed to revert this decrease back to 137 ± 18% and 157 ± 30% (p < 0.05), respectively.

### Liver-kidney dysfunction indices

Table 1 illustrates that the activities of AST, ALT and GGT in the plasma of the diabetic rats underwent significant (p < 0.05) increases of up to 242 ± 35%, 301 ± 35%, and 296 ± 29% respectively, when compared to non-diabetic rats. The administration of kombucha or black tea was found to bring about marked decreases in terms of the three indices of liver toxicity. Moreover, and when compared to non-diabetic rats, the diabetic rats were noted to undergo significant (p < 0.05) increases of 203 ± 17% and 196 ± 15% in terms of the urea and creatinine rates in the plasma, respectively. Interestingly, the administration of kombucha and black tea to diabetic rats was observed to have reverted this increase back to normal. The findings from histological analyses further confirmed the positive effect of those two supplements on the liver and kidney (data not shown).

**Table 1:** Effect of KT and BT on liver-kidney dysfunction indices in plasma of surviving diabetic rats

	**Con**	**Con + BT**	**Con + KT**	**Diab**	**Diab + BT**	**Diab + KT**
***Liver dysfunction indices***						
AST (U/L)	32 ± 2.37^*^	31.67 ± 3.56^*^	29.5 ± 3.02^*^	77.17 ± 9.35	52.58 ± 23.75	47.75 ± 36.14
ALT (U/L)	24.25 ± 2.79^*^	23.33 ± 4.13^*^	21.33 ± 4.13^*^	72.33 ± 5.47	41.67 ± 5.16^*@^	36.9 ± 10.68^*@^
GGT (U/L)	15.67 ± 2.73^*^	16.50 ± 4.76^*^	13.67 ± 1.63^*^	45.83 ± 6.15	26.83 ± 6.40^*@^	23.67 ± 5.88^*^
***Kidney toxicity indices***						
Creatinine (mg/L)	13.02 ± 1.42^*^	12 ± 2.19^*^	9.83 ± 1.47^*^	25.5 ± 2.59	18.83 ± 2.86^*@^	15.4 ± 2.59^*^
Urea (g/L)	0.52 ± 0.07^*^	0.5 ± 0.09^*^	0.47 ± 0.06^*^	1.05 ± 0.11	0.77 ± 0.36^*^	0.63 ± 0.25^*^

Data represent mean ± S.D (n = 8 for each group). The values are statistically presented as follows: single asterisk (*), p < 0.05 vs. control diabetic rats (Diab); commercial at (@), p < 0.05 vs. respective control rats (Con) [i.e. on the same corresponding tea treatment].

## Discussion

Although kombucha tea is popular around the world as a beneficial medicinal health-promoting drink, its beneficial and/or adverse effects on human health have not been scientifically determined yet. No previous study has, for instance, so far reported on the systematic investigation and evaluation of the antidiabetic activity of kombucha. To the authors’ knowledge, the present work is the first attempt to investigate the protective effects of kombucha on diabetes and its complications on the functions of the liver, kidney, and pancreas.

Several of the enzymes secreted by the pancreas, namely α-amylase and lipase, are known to break down dietary polysaccharides and lipids into monosaccharides and free fatty acids, which represent some of the major nutrients needed to maintain human health [36,37]. Although most of the research so far conducted on diabetes has focused on dyslipidemia as a major risk factor for cardiac, cerebral, and renal complications, several studies have recently showed an impairment of pancreatic exocrine function in type 1 and type 2 diabetes. The analysis of serum/plasma pancreatic enzymes was suggested to provide additional informative parameters for the assessment of the chronicity and progress of the illness as well as of the response to therapy [38-41].

The findings of the present study showed that the administration of kombucha to surviving diabetic rats significantly reduced pancreatic α-amylase activity, which plays a key role in the digestion of carbohydrates. This was indicative of lowered levels of absorbable glucose being formed from the digestion of carbohydrate and leading to reduced levels of blood glucose. The inhibition of pancreatic α-amylase activity in the human digestive tract represents one of the therapeutic approaches commonly used for the control and prevention of postprandial hyperglycemia in non-insulin-dependent diabetic patients through reducing the uptake of glucose released by those enzymes from starch [42,43].

To produce kombucha, black tea ingredients and sucrose undergo progressive modifications due to the action of the tea fungus. Several metabolites can be identified in the fermented beverage, including acetic, lactic, gluconic and glucuronic acids, ethanol, glycerol and polyphenols [20,44-46]. Most of the properties of kombucha are attributed to the polyphenolic composition of the beverage. Tea polyphenolics were previously reported to inhibit and reduce α-amylase activity in the in the saliva and intestines of rats, respectively, which, in turn, were described to lower the hydrolysis of starch to glucose and to reduce the assimilation of glucose [47].

The present study also showed that the administration of kumbucha to surviving diabetic rats reduced pancreatic lipase activity, a decrease that is responsible for the hydrolysis of non-absorbable dietary triglycerides into absorbable monoglycerides and free fatty acids, which, in turn, leads to the decrease of plasma cholesterol and TG level [48-50]. This represents one of the therapeutic approaches commonly used for the control and prevention of dyslipidemia. Polyphenols were reported to inhibit pancreatic lipase *in vitro*. Previous data suggested that the presence of galloyl moieties within polyphenol chemical structures was required for the enhancement of pancreatic lipase inhibition [51].

Alloxan is a specific toxin that destroys the pancreatic β-cells, provoking a state of primary deficiency in insulin without affecting other types of islets. The diabetic effect of alloxan is due to an excess in the production of reactive oxygen species (ROS). This excess leads to toxicity in pancreatic cells, which, in turn, reduces the synthesis and release of insulin while concurrently affecting other organs, such as liver [52]. Increased lipid peroxidation products and decreased plasma or tissue concentrations of superoxide dismutase, catalase, and glutathione have been well documented in the literature on alloxan-induced diabetes [30,53].

Chronic hyperglycemia and dyslipidemia are associated with a variety of metabolic disorders in human and animal diabetic patients [54,55], causing oxidative stress, depleting the activity of the antioxidative defense system, and resulting in elevated levels of ROS [30,43]. Oxidative environments might cause the damage of cells and tissues in the liver and kidney [5], which is observed in the increased levels of AST, ALT, and GGT activities (indices of liver dysfunction) and of urea and creatinine (indices of kidney dysfunction). As far as the present study is concerned, the findings showed that kombucha proved remarkably efficient in the decrease of the liver and kidney dysfunction indices in surviving diabetic rats, namely the AST, ALT, and GGT activities and the urea and creatinine levels. This supplement could, therefore, be considered as a potential strong candidate for future industrial application as a therapeutic agent against liver and kidney toxicity.

Several recent studies have provided ample support for the strong candidacy of kombucha for application as an antioxidant agent for the alleviation of oxidative stress and free radicals as well as the enhancement of enzymatic defenses. Bhattacharya *et al.* have, for instance, showed that murine hepatocytes treated with KT prevented the disruption of mitochondrial membrane potential and blocked the activation of mitochondria-dependent apoptotic signaling pathways, thus displaying a significant reduction of tertiary butyl hydroperoxide-induced ROS generation and a considerable attenuation of malonaldehyde levels [20]. The inhibition of radical species could, therefore, be one of the mechanisms involved in the efficient hepatoprotective and curative properties of KT. These findings are, in fact, in good agreement with the results previously reported by Murugesan *et al.* showing that KT has the potential to revert the CCl_4_-induced hepatotoxicity back through the production of antioxidant molecules during fermentation [17]. The presence of glucaric acid and its derivatives, as potent detoxifying agents, could also be considered as another reason for the hepatoprotective effects of KT [19].

Furthermore, Gharib *et al.* showed that, owing to its antioxidant potential, KT can ameliorate trichloroethylene-induced kidney damage by preventing lipid peroxidation and ROS species formation [16]. The nephroprotective effects kombucha were also attributed to organic acids (e.g. acetic and glucuronic acids) which are known to facilitate the detoxification process through conjugation with toxins, which they then solubilise and eliminate from the body [56].

Kombucha polyphenols may, therefore, prevent the damage and death of pancreatic β-cells, and/or stimulate the regeneration of this type of cells in diabetic rats. Coskun *et al.* have reported that the administration of polyphenols, such as quercetin and epicatechin, to surviving diabetic rats protects the architecture of pancreatic β-cells, preserves the secretion of insulin, and stimulates the regeneration of this type of cells [57]. The administration of an antioxidant-rich beverage, such as kombucha, to diabetic rats would, therefore, presumably decrease the ROS-mediated toxicity in pancreatic β-cells [35]. The ability of kombucha to reduce the blood glucose level could also be attributed to its ability to modulate the immune system [18], leading to the decrease of β-cell damages. It is worth noting that the findings indicated that the curative effects achieved with the administration of KT were more pronounced than those reported for BT, which could presumably be attributed to the large amounts of polyphenols and flavonoids present in KT as compared to black tea [20]. In fact, further studies on the mechanisms and modes of action of kombucha are needed to fully appreciate its values and limitations.

## Conclusions

Overall, the present study demonstrated that the hypoglycemic and antilipidemic activities exhibited by kombucha tea were effective enough to alleviate alloxan-induced diabetes in experimental rats. The beneficial effect of dietary kombucha is presumably attributed to its potent hypoglycemic and antilipidemic properties, as well as antioxidant potential. Further studies are obviously needed to capitalize on the protective effects of kombucha tea in humans and to make its use suitable as an effective functional food with therapeutic potential. The findings presented in this work are encouraging and substantiate the search for newer pharmacophores in kombucha behind the hypoglycemic and antilipidemic effects. The possible mechanisms underlying the inhibition of pancreatic α-amylase and lipase are also yet to be studied.

## Abbreviations

KT: Kombucha tea; BT: Black tea; AST: Aspartate transaminase; ALT: Alanine transaminase; GGT: Gamma-glytamyl transpeptidase; HDL-Ch: High-density lipoprotein-cholesterol; LDL-Ch: Low-density lipoprotein-cholesterol; TG: Triglycerides; ROS: Reactive oxygen species; DM: Diabetes mellitus.

## Competing interests

The authors declare that they have no competing interests.

## Authors’ contributions

All authors participated in the conception, set up, and carrying out of the study, as well as in the handling and interpretation of its findings, and the writing and reviewing of its text. AA and KH have equally contributed to this work and undertook data analysis. DE, MB and KH contributed to the interpretation of the data. BJ helped in the writing and reviewing process. FA contributed in the design of the study and the discussion of the data. EA and AE supervised the work. All authors have read and approved of the final manuscript.

## Pre-publication history

The pre-publication history for this paper can be accessed here:

http://www.biomedcentral.com/1472-6882/12/63/prepub
